# Efficacy and safety of surgery in renal carcinoma patients 75 years and older: a retrospective analysis

**DOI:** 10.1186/s12894-022-01088-3

**Published:** 2022-08-29

**Authors:** Hongsong Bai, Weixing Jiang, Dong Wang, Jianzhong Shou, Changling Li, Nianzeng Xing

**Affiliations:** 1 Department of Urology, National Cancer Center/National Clinical Research Center for Cancer/Cancer Hospital, Chinese Academy of Medical Sciences and Peking Union Medical College, Beijing, 100021 China; 2 Department of Urology, Cancer Hospital of HuanXing, ChaoYang District, Beijing, 100023 China; 3 Department of Urology, Beijing Friendship Hospital, Capital Medical University, Beijing, 100050 China

**Keywords:** Renal cell carcinoma, Elderly, Complications, Partial nephrectomy

## Abstract

**Objective:**

To investigate the efficacy and complications of surgical treatment in patients with renal cell carcinoma aged ≥ 75 years.

**Methods:**

From January 2009 to May 2019, we assessed 166 patients aged 75 years and older, who either had radical nephrectomy (RN) or partial nephrectomy (PN) as treatments for diagnosed renal cell carcinoma. Patients were divided into one group of patients aged 75–79 years and the second group of patients ≥ 80 years. The complications and survival were compared between the two groups.

**Results:**

All 166 patients were successfully operated on. Differences between the two groups were statistically significant in intraoperative and postoperative complications and Clavien–Dindo score of ≥ 1 (*P* = 0.02, *P* < 0.001, *P* = 0.001). Univariate analysis revealed no significant correlation between a Clavien–Dindo score ≥ 1 versus gender, body mass index (BMI), lack of symptoms, KPS, baseline GFR, postoperative GFR, tumor size, tumor location, surgical method, and transfusion or no transfusion (ALL *P* > 0.05). Multifactor analysis showed that age ≥ 80 years, partial nephrectomy, and operation time were independent predictors of a Clavien–Dindo score ≥ 1. No significant difference was found in OS between the two groups, (*P* < 0.0001), and no significant difference in CSS (*P* = 0.056). There was no significant difference in OS and CSS between the RN and PN groups (*P* = 0.143, *P* = 0.281, respectively).

**Conclusions:**

According to our findings, the overall safety of surgical therapy for elderly patients with renal cell carcinoma is adequate. PN should be carefully examined, especially over the age of 80. To select suitable patients based on an assessment of the tumor's complexity and patients' physical condition, such as age, underlying diseases and other conditions, technical feasibility, balance of benefits and a case-by-case.

## Introduction

Renal cell carcinoma accounts for around 4.0% of all malignant tumors in the body. In 2021, there were nearly 76,080 new cases in the United States, suggesting an increasing trend year by year [1]. Similarly, the incidence of renal cell carcinoma in China also showed a gradually increasing trend, and the incidence increased with advancing age [2]. Surgery is the primary treatment for localized renal cancer. However, for elderly patients, due to multiple underlying diseases, there is an increased surgical risk and a risk of postoperative complications. In 2017, the American Society of Clinical Oncology (ASCO) recommended active surveillance as the preferred treatment for patients with small renal cell carcinoma with high risk factors and poor life expectancy. The scope of application and absolute indications (high risk of surgical anesthesia or life expectancy < 5 years) have been clarified [3]. However, some studies have shown that elderly patients benefit from surgery, and partial nephrectomy can be used as an alternative. Therefore, there is still controversy about the benefits of surgical treatment in elderly patients. Screening should be carried out according to the condition and circumstance of patients, in order to better choose a more optimized treatment plan, improve therapeutic efficacy, and reduce complications. The results of our study support this.

The Clavien–Dindo classification (CDC) is a method for systematically recording surgical complications. Clavien developed it in 1992, and Dindo et al. modified it in 2004 in order to increase its accuracy and acceptability in clinical practice [4, 5] (Table 1). The CDC technique has been verified and recognized for use in a range of surgical specialties throughout the world [6, 7]. Its major characteristic is that the severity of a complication is evaluated based on the type of therapy required to resolve the issue. According to the CDC system, this study discusses variations in complications across age groups in elderly patients, as well as associated factors.

**Table 1:** Clavien–Dindo classification of surgical complications

Grade	Definition
Grade I	Any deviation from the normal postoperative course without the need for pharmacological treatment or surgical, endoscopic, and radiological interventions allowed therapeutic regimens are: drugs as antiemetics, antipyretics, analgetics, diuretics, electrolytes, and physiotherapy. This grade also includes wound infections opened at the bedside
Grade II	Requiring pharmacological treatment with drugs other than such allowed for grade I complications Blood transfusions and total parenteral nutrition are also included
Grade III	Requiring surgical, endoscopic or radiological intervention
IIIa	Intervention not under general anesthesia
IIIb	Intervention under general anesthesia
Grade IV	Life-threatening complication (including CNS complications)* requiring IC/ICU management
IVa	Single organ dysfunction (including dialysis)
IVb	Multiorgan dysfunction
Grade V	Death of a patient

*CNS* central nervous system, *IC* intermediate care, *ICU* intensive care unit

*Brain hemorrhage, ischemic stroke, subarachnoid bleeding, but excluding transient ischemic attacks

## Materials and methods

### Patient selection and data extraction

A retrospective analysis was performed on the clinical data of 166 patients who aged ≥ 75 years at the time of surgery were consecutively enrolled with localized renal cell carcinoma, derived from the medical record system, the Department of Urology, Cancer Hospital, Chinese Academy of Medical Sciences from February 2009 to February 2019.

According to the European Association of Urology(EAU), National Comprehensive Cancer Network (NCCN), and ASCO guidelines [3, 8, 9], for patients with small renal tumors (SRM), active surveillance (AS), surgical treatment (PN or RN), thermal ablation (TA) are feasible, and recommended for elderly patients and less than 3 cm or 4 cm and the tumor growth rate is less than 0.5 cm/year, high risk and feasible active monitoring of life expectancy of less than 5 years, but the treatment methods acceptable to both doctors and patients should be established according to the characteristics of the tumor and related factors of the patients themselves, but in China there is no large sample AS and TA of research data, and also has the research shows that AS and TA compared with the surgical treatment, CSS and OS are not benefit [10], and in patients with SRM, based on the recommendations of guidelines, we will explain in detail the research data and advantages and disadvantages of AS, RN,PN and TA, and finally patients will decide the treatment method. However, due to the fear and tension after the discovery of the tumor, patients will choose more active surgical treatment.

The study cohort included 111 men and 55 women, with an age range from 75 to 89 years. The clinical data included general information such as age, gender, tumor size, surgical method, imaging, and pathology. Patients were divided into one group of patients aged 75–79 years and the second group of patients ≥ 80 years. The method of operation through a retroperitoneal approach was laparoscope or open surgery, general anesthesia, and lateral decubitus position. All the operations were completed. The patient's vital signs and drainage were closely observed after surgery, and the patients were advised to rise early in the morning and resume their diet. The intraoperative and postoperative complications were compared between the two groups, and related factors were analyzed according to the CDC.

### Preoperative assessment methods

According to the Chinese Experts' Recommendations for Preoperative Assessment of Elderly Patients (2015), physical condition assessment should be conducted first, including assessments of frailty of state, functional/physical state and fall risk, cognitive dysfunction, mental state, cardiac and pulmonary complication risk. Additional assessments include risk of stroke, renal function, thrombosis, bleeding risk, and nutritional status. Based on the assessment results, active medication and other treatments to control complications could be provided. Multi-Disciplinary Treatment (MDT) rounds were conducted before the operation, and the planned operation plan was formulated. The patients and their families were informed of the planned treatment plan or alternative plan, and the patients and their families were informed of the condition and signed informed consent.

### Follow-up methods

All patients were reviewed regularly from 3 to 6 months, including chest X-ray, abdominal and pelvic ultrasound, blood routine examination, liver and kidney function. Abdominal and pelvic CT or abdominal MRI were reviewed every 6 months, and chest CT was reviewed after no more than one year. Regular follow-up was conducted by telephone and medical record inquiry system.

### Statistical methods

IBM SPSS Statistics for Windows 20.0 was used for statistical analysis (IBM, Armonk, NY, USA). For measurement data with a normal distribution, the mean ± SD was used, and the median and range were used in a non-normal distribution. The Student's t-test was used to examine quantitative variables. The chi-squared test, Fisher's exact test, and Mann–Whitney U test were used to assess categorical data. Furthermore, to evaluate risk factors for the Clavien–Dindo score ≥ 1 variables were included in the univariate analysis using Fisher's exact test. Univariate variables with a *P* value less than 0.10 were then included in multivariate logistic regression analysis. The OS and CSS rates were subjected to a Kaplan–Meier survival analysis. GraphPad Prism V9.0.0 was used for statistical drawing. Differences with a *P* value of less than 0.05 were considered statistically significant.

## Results

### Population characteristics

The data of patients aged 75–79 years and ≥ 80 years are summarized in Table 2. There were statistical differences in age, KPS score status, number of underlying diseases, baseline glomerular filtration rate (GFR) and postoperative GFR between the two groups (ALL *P* < 0.05). There were no significant differences in gender, BMI, symptoms at initial diagnosis, median tumor size and location, surgical type, surgical method, pathological type, ISUP grade, RENAL score, lymph node metastasis, and pathological stage between the two groups (*P* > 0.05). This suggests that the general status of the ≥ 80 years group was slightly worse than that of the aged 75–79 years group.

**Table 2:** Patient clinical and pathologic characteristics

Characteristics	Total (n = 166)	Age 75–79 (n = 130)	Age ≥ 80 (n = 36)	*P* value
Age at surgery (median)	77 (75–89)	76 (75–79)	82 (80–89)	< 0.001
Gender, n (%)				0.907
Man	112 (67.5)	88 (67.7)	24 (66.7)	
Woman	54 (32.5)	42 (32.3)	12 (33.3)	
BMI (mean ± SD)	21.5 ± 1.8	21.3 ± 2.5	21.6 ± 1.6	0.536
KPS score < 80, n (%)	23 (13.9)	15 (11.5)	8 (22.2)	0.016
Number of underlying diseases, n (%)				0.019
< 2	106 (63.9)	89 (68.5)	17 (47.2)	
≥ 2	60 (36.1)	41 (31.5)	19 (52.8)	
Presenting symptom, n (%)	25 (15.1)	19 (14.6)	6 (16.7)	0.761
Baseline eGFR (ml/min)	70.5 ± 9.7	72.1 ± 5.9	63.1 ± 6.4	< 0.001
Postoperative eGFR (ml/min)	61.7 ± 8.8	63.3 ± 5.4	52.1 ± 5.6	< 0.001
Tumor size (median)	5.1 (1.8–13)	5.0 (2.5–12.6)	5.3	0.314
Tumor location, n (%)				0.831
Left	85 (51.2)	66 (50.8)	19 (52.8)	
Right	83 (48.8)	64 (49.2)	17 (47.2)	
Surgery procedure, n (%)				0.772
Open	12 (7.2)	9 (6.9)	3 (8.3)	
Laparoscopic	154 (92.8)	121 (93.1)	33 (91.7)	
Surgery method				0.097
Radical	115 (69.3)	86 (66.2)	29 (80.6)	
Partial	51 (30.7)	44 (33.8)	7 (19.4)	
RENAL score (partial)				0.471
≤ 9	39 (76.5)	33 (75.0)	6 (85.7)	
> 9	12 (23.5)	11 (25.0)	1 (14.3)	
Pathological type				0.960
Clear cell carcinoma	144 (86.7)	113 (86.9)	31 (86.1)	
Papillary	11 (6.6)	8 (6.2)	3 (8.3)	
Chromophobe	6 (3.6)	5 (3.8)	1 (2.8)	
Others	5 (3.0)	4 (3.1)	1 (2.8)	
ISUP grade, n (%)				0.595
Low grade ≤ 2	95 (57.2)	73 (56.2)	22 (61.1)	
High grade ≥ 3	71 (42.8)	57 (43.8)	14 (38.9)	
Tumor necrosis, n (%)	29 (17.5)	21 (16.2)	8 (22.2)	0.724
Sarcomatoid differentiation, n (%)	26 (15.7)	19 (14.6)	7 (19.4)	0.481
Lymph node metastasis, n (%)	8 (4.8)	6 (4.6)	2 (5.6)	0.816
Pathological T stage, n (%)				0.794
T1	134 (80.7)	105 (80.8)	29 (80.6)	
T2	21 (12.7)	17 (13.1)	4 (11.1)	
T3	9 (5.4)	7 (5.4)	2 (5.6)	
T4	2 (1.2)	1 (0.8)	1 (2.8)	

The method of operation through a retroperitoneal approach was laparoscope or open surgery, general anesthesia, lateral decubitus position. Among the 166 patients, 115 were treated with radical nephrectomy, 51 with partial nephrectomy, 12 with open surgery, 154 with laparoscopic surgery, three with intraoperative blood transfusion, and three with pleural injury and peripheral organ injury. The remaining patients had no obvious intraoperative complications, and all the operations were completed. The patient's vital signs and drainage were closely observed after surgery, and the patients were advised to rise early in the morning and resume their diet.

### Comparison of perioperative complications between the two groups

Comparisons of postoperative complications between aged 75–79 years and ≥ 80 years groups using the nonstandardized evaluation system and CDC system are shown in Table 3. There were no significant differences in intraoperative blood loss and operation time between the two groups (*P* > 0.05), while there were significant differences in in-hospital stay, perioperative 90-day mortality, and Clavien–Dindo scores between the two groups (*P* < 0.001).

**Table 3:** Perioperative complications in elderly patients

	Age 75–79 (n = 130)	Age ≥ 80 (n = 36)	*P* value
**Intraoperative complications, n (%)**	**3 (2.3)**	**3 (8.3)**	**0.020**
Blood transfusion, n (%)	2 (1.5)	1 (2.8)	0.621
Inferior vena cava injury, n (%)	0 (0)	0 (0)	–
Pleural injury, n (%)	0 (0)	1 (2.8)	0.057
Pulmonary embolism, n (%)	0 (0)	0 (0)	–
Intraoperative death, n (%)	0 (0)	0 (0)	–
Organs injury, n (%)	1 (0.8)	1 (2.8)	0.328
Mean estimated blood loss, ml (mean ± SD)	97.3 ± 49.5	94.7 ± 40.6	0.714
Operative time, mins (mean ± SD)	118.0 ± 45.1	112.1 ± 15.7	0.737
**Postoperative complications, n (%)**	**8 (6.2)**	**10 (27.8)**	**< 0.001**
Wound healing disorder, n (%)	3 (2.3)	2 (5.6)	0.313
Infections, n (%)	1 (0.8)	2 (5.6)	0.056
Postoperative hemorrhage, n (%)	1 (0.8)	0 (0)	0.598
Gut obstruction, n (%)	1 (0.8)	1 (2.8)	0.328
Acute kidney injury, n (%)	0 (0)	0 (0)	–
Pulmonary embolism, n (%)	0 (0)	0 (0)	–
Congestive heart failure, n (%)	1 (0.8)	3 (8.3)	0.009
Deep vein thrombosis, n (%)	1 (0.8)	2 (5.6)	0.056
Median length of stay, days (mean ± SD)	8.7 ± 2.3	12.0 ± 5.6	< 0.001
Perioperative mortality within 90 days, n (%)	2 (1.5)	3 (8.3)	0.035
Clavien−Dindo score			0.001
0	37 (28.5)	4 (11.1)	
1–2	91 (70.0)	27 (75.0)	
≥ 3	2 (1.5)	5 (13.9)	

There were three intraoperative complications in the aged 75–79 years group, including two cases of blood transfusion and one case of peripheral organ injury. There were three complications in the ≥ 80 years group, including one case of blood transfusion, one case of pleural injury, and one case of peripheral organ injury. Intraoperative complications differed significantly between the two groups (*P* < 0.05).

There were eight cases of postoperative complications in the aged 75–79 years group, including poor wound healing in three patients, postoperative infection in one case, postoperative bleeding in one case, intestinal obstruction in one case, congestive heart failure in one case, and deep vein thrombosis in one case. In the ≥ 80 years group, there were postoperative complications in 10 cases, including poor wound healing in two cases, postoperative infection in two cases, intestinal obstruction in one case, congestive heart failure in three cases, and deep vein thrombosis in two cases. Postoperative complications differed significantly between the two groups (*P* < 0.05).

A total of 125 patients (75.3%) had complications Clavien–Dindo score ≥ 1. Univariate analysis revealed that the factors significantly associated with an increased risk of complications Clavien–Dindo score ≥ 1 were advanced age (≥ 80 years), Number of underlying diseases (≥ 2), type of surgery (PN), and longer operative time. Multivariate logistic regression analysis of these factors showed that age (≥ 80 years), partial nephrectomy, and longer operation time were independent risk factors for postoperative complications Clavien–Dindo score ≥ 1 (Table 4).

**Table 4:** Univariate and multivariate Cox analysis of risk factors associated with Clavien–Dindo score ≥ 1

	Univariate			Multivariate		
	OR	95% CI	*P* value	OR	95% CI	*P* value
Age (≥ 80)	7.286	1.667–31.8	0.008	15.394	1.531–35.753	0.020
Gender (male)	0.594	0.266–1.32	0.203		–	
BMI, kg/m^2^	0.914	0.745–1.12	0.391		–	
Presenting symptom	1.046	0.387–2.82	0.930		–	
Number of underlying diseases (≥ 2)	2.938	1.255–6.87	0.013	1.101	0.229–5.301	0.905
KPS (< 80)	1.371	0.480–3.92	0.556		–	
GFR, ml/min	0.989	0.954–1.02	0.572		–	
GFR, ml/min (postoperative)	0.878	0.786–0.99	0.489		–	
Tumor size, cm	0.924	0.750–1.13	0.461		–	
Type of surgery (partial nephrectomy)	5.574	1.868–16.6	0.002	3.193	1.003–10.934	0.046
Tumor side (left)	1.295	0.639–2.62	0.473		–	
Surgery procedure (open)	0.983	0.253–3.81	0.980		–	
Operative time, min	1.075	1.046–1.10	< 0.001	1.096	1.058–1.136	< 0.001
Intraoperative blood transfusion (yes)	0.650	0.057–7.36	0.728		–	
Intraoperative estimated blood loss, ml	1.003	0.995–1.01	0.438		–	

*BMI* body mass index, *KPS* Karnofsky performance status, *GFR* glomerular filtration rate

### Comparison of survival between the two groups and RN and PN

Overall survival—After a median followup of 45.8 months (IQR: 6.4–129.9), 44 patients died (26.5%).

**Fig. 1:**
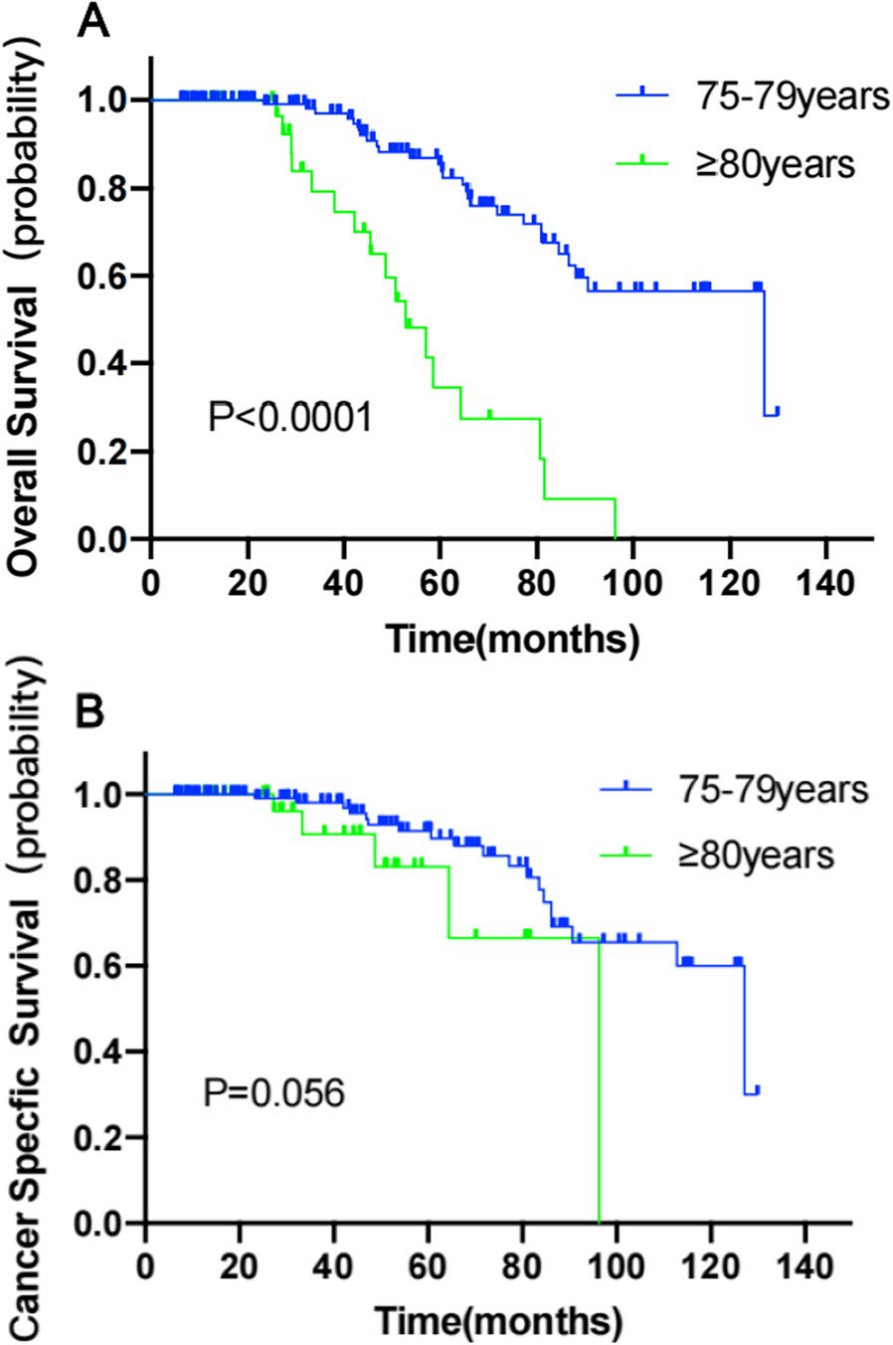
Kaplan–Meier estimates depicting the overall survival (**A**), cancer-specifific survival (**B**) stratifified according to age 75–79 years [blue curves] versus ≥ 80 years [green curves])

OS at 3 and 5 years were 96.9% and 85.5% in the 75–79 years group, 79.4% and 34.4% in the ≥ 80 years group, respectively. Significant difference was found between the two groups (HR 0.20, 95% CI 0.08–0.51, *P* < 0.0001) (Fig. 1A).

**Fig. 2:**
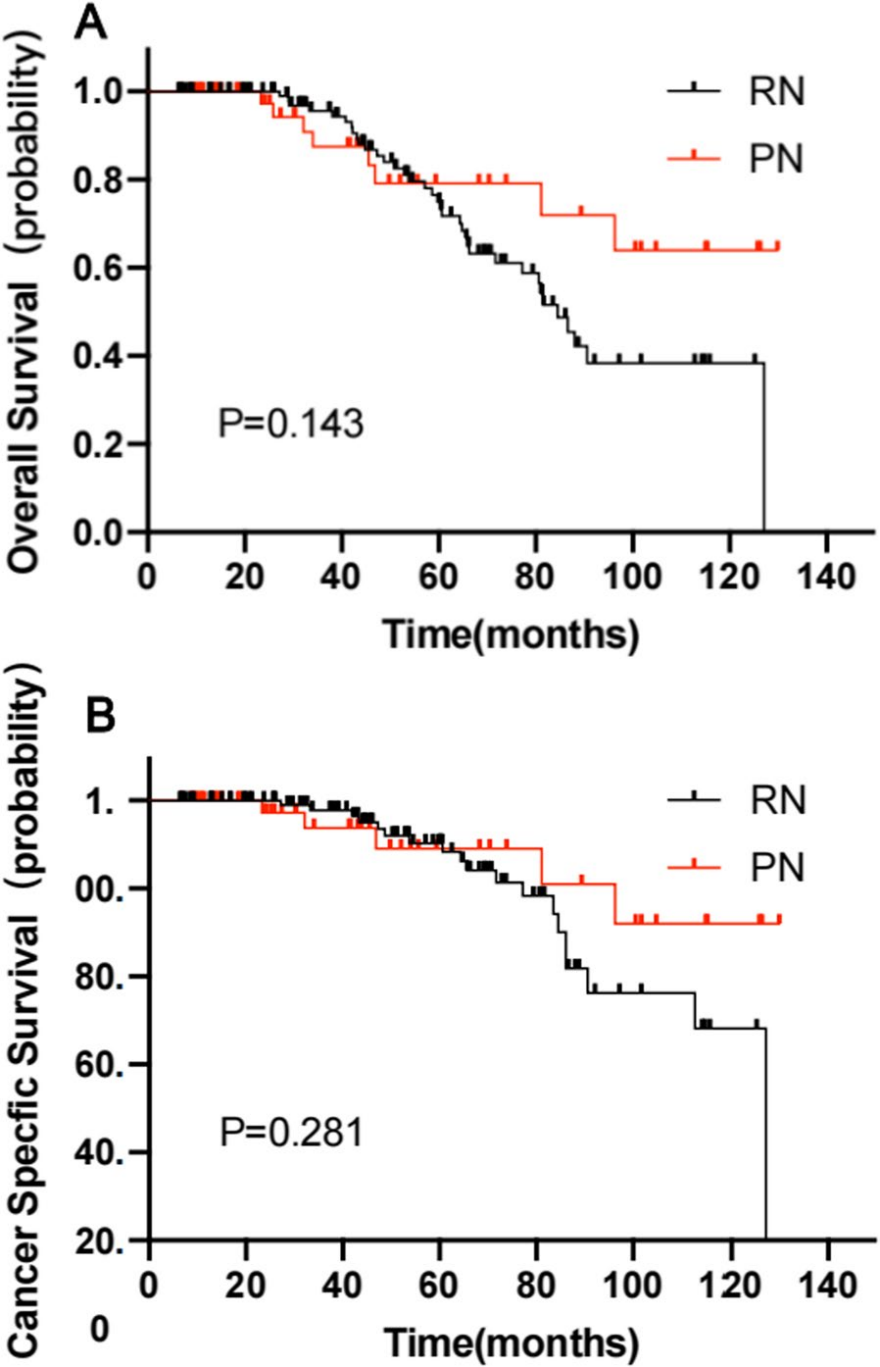
Kaplan–Meier estimates depicting the overall survival (**A**), cancer specific survival (**B**), stratifified according to treatment type (partial nephrectomy [PN, red curves] versus radical nephrectomy [RN, black curves])

OS at 3 and 5 years were 95.5% and 74.9% in the RN group, 87.5% and 79.1% in the PN group, respectively. No Significant difference was found between the two groups (HR 1.65, 95% CI 0.84–3.21, *P* = 0.143) (Fig. 2A).

Cancer specific survival—There were 20 deaths (12.0%) related to cancer.

CSS at 3 and 5 years were 98.0% and 91.4% in the 75–79 years group, 90.8% and 83.1% in the ≥ 80 years group, respectively. Although there was a difference between the two groups, no significant difference was found (HR 0.26, 95% CI 0.06–1.04, *P* = 0.056) (Fig. 1B).

CSS at 3 and 5 years were 97.7% and 90.3% in the RN group, 93.8% and 89.1% in the PN group, respectively. No significant difference was found (HR 1.63, 95% CI 0.67–3.93, *P* = 0.281) (Fig. 2B).

## Discussion

For localized renal cancer, major guidelines recommend surgical treatment [8, 9], including radical nephrectomy and partial nephrectomy, as well as active monitoring or radiofrequency ablation. Nephron-sparing surgery is preferred because of its advantages in protecting renal function [9]. In 2006, partial nephrectomy became the gold standard in the treatment of stage T1 tumors [11]. According to China's seventh population census, the number of people aged 60 or over was 264.02 million, accounting for 18.7%. This was an increase of 5.44% compared with 2010, indicating that the degree of aging has further deepened. In addition, the proportion of kidney cancer in the elderly population has further increased.

Although EORTC 30904 [12] suggested that patients undergoing radical nephrectomy had improved survival, some studies supported elderly patients undergoing nephron-sparing surgery [13]. Thus, the advantages of the two surgical methods remain controversial. For elderly patients, it is not clear how to choose between the two surgical methods and whether partial nephrectomy is beneficial [14–16]. Therefore, the choice of surgical method depends on patient characteristics and the functions of various organs [17], especially for patients over 75 years old. Additionally, the expected survival of patients and the possible complications caused by surgery that may affect the quality of life of patients should also be assessed.

Previous studies suggest that there are more complications associated with basic diseases, such as hypertension and heart disease, in elderly patients over the age of 70. However, there were no significant differences in operative time, blood loss and perioperative complications between patients under the age of 70 and patients undergoing laparoscopic radical nephrectomy. The incidence of intraoperative complications was 2.9% and 5.3%, respectively, and the incidence of postoperative complications was 8.8% and 4.2%, respectively. In comparison, the incidence of postoperative complications was higher in patients over 70 years old of age [18].

Berdjis et al. [19] conducted a study on 115 renal cancer patients over 75 years old and 908 patients under 75 years old who received surgical treatment. The finding was that age is not a contraindication for surgery. Overall complications and mortality of patients over 75 years old showed no significant difference compared with those under 75 years old, but most of them were radical nephrectomy, and partial nephrectomy accounted for only 13.4%. Staehler et al. [20] analyzed 117 patients with renal cancer who underwent surgical treatment and found that the incidence of perioperative complications of partial nephrectomy and radical nephrectomy was 12% and 15%, respectively, and the incidence of complications within 30 days was 4% and 7%, with no significant difference. However, Sun et al. [21] found that there were more complications in elderly patients over 75, and death was mostly due to cardiovascular and cerebrovascular diseases and non-tumor causes. Many studies support nephron-sparing surgery for elderly patients [13].

It should be noted that most of the previous studies were analyzed by age grouping at the age of 75 or by surgical grouping. In particular, there are few stratified studies on elderly patients aged over 75. The current study collected the data of 166 patients over 75 years old in our hospital and analyzed the general information, complications, and survival.

There was a substantial statistical difference in preoperative baseline eGFR between the two groups, which was thought to be due to age, There was also a significant difference in postoperative GFR (reexamination within 1 week following surgery) between the two groups. All patients' postoperative eGFR decreased, and as compared to the 75–79 years group, the ≥ 80 years group experienced a higher percentage drop in eGFR from baseline. Phillip et al. [22] and Rivero et al. [23] discovered that RN is related to worse renal outcomes than PN. Another study [24] confirmed that eGFR loss related to renal cancer surgery, whether due to PN or RN, increases the risk of chronic kidney disease but has a lesser influence on survival. In our study, there were no cases of chronic renal disease or fatalities from it in either group, which may be connected to the larger number of patients undergoing RN in this group. We believe that baseline renal function and age, which reflect general health conditions, can predict long-term renal functional results independent of surgery type. Therefore, a patient with a large tumor and chronic kidney disease may benefit from PN.

Our study found that the 75–79 years group had superior survival to the ≥ 80 years group (*P* < 0.0001). The results were similar to the previous studies [13, 25, 26]. This is mainly associated with older age, poor basic conditions, lower overall life expectancy, and a higher proportion of deaths from non-tumor mortality. In the ≥ 80 years group, a total of 17 patients died, 12 (70.6%) died of non-tumor causes, in the 75–79 years group, a total of 27 patients died, 12 (44.4%) died of non-tumor causes. And there was no significant difference was found in CSS between the two groups, although it was greater in the 75–79 years group, which may be related to the cause of death (P = 0.056).

Although most results suggest that OS is better with PN than with RN [27–30], similarly, most studies have shown that PN and RN have similar OS in patients, PN was not beneficial in terms of OS in elderly patients (≥ 65 years old), the 5-year OS rates after surgery were 94.7% for PN versus 91.9% for RN (*P* = 0.698) [13, 31]. And no significant difference was found (*P* = 0.281) in CSS in our study. Several previous studies have also found similar or opposite results. The Meta-Analysis (a total of 60 studies) showed that CSS estimates among all management strategies were 95 to 100% and did not differ significantly among treatments. Comparative analyses of RN and PN indicated that increasing age, larger tumor size, and higher tumor grade were the most common predictors of worse CSS [32]. However, some studies showed no difference in CSS between RN and PN when stratified by age, tumor size or grade [33–35]. Further confirmation is needed from larger samples and prospective controlled studies.

Using the Clavien–Dindo classification method, postoperative complications were defined as complications occurring within 30 days after surgery [5]. In our study, the overall complication rate was 14.45%, it was higher than that in previous studies, which may be related to the age composition of patients and the choice of surgical methods. In our study, patients aged ≥ 80 years accounted for 21.7%, and partial nephrectomy accounted for 30.7%. However, most of the previous studies focused on radical nephrectomy. The results of our study suggest that partial nephrectomy should be fully evaluated for patients ≥ 80 years of age. Although studies have shown a gradual increase in the use of partial nephrectomy in patients ≥ 65 years of age (41% in patients over 75 and 14.9% in patients over 80), the increased application rate was not significantly correlated with the presence of concomitant diseases such as heart disease (*P* = 0.256), kidney disease (*P* = 0.419), diabetes (*P* = 0.808), and hypertension (*P* = 0.931); thus patients could benefit from nephron-sparing surgery [36]. However, that study only analyzed the application of partial nephrectomy in elderly patients in recent years and its impact on survival without summarizing the perioperative complications. Our study suggests partial nephrectomy application showed more complications in elderly patients, especially in patients aged ≥ 80 years with a lengthy hospital stay, with higher mortality. In addition, the findings of Chung et al. suggested that partial nephrectomy did not significantly prolong the survival of elderly patients [13].

Therefore, for patients over the age of 75, and especially for those aged ≥ 80, partial nephrectomy should be carefully selected. Further data collection is needed to verify whether partial nephrectomy is beneficial to prolong survival. However, the purpose of our study was not to reduce the use of partial nephrectomy in elderly patients but to select suitable patients based on clinical characteristics. The surgical risk prediction model can be used for preoperative risk prediction just like the Model of the American College of Surgeons to carry out precision surgical treatment [37]. Due to the retrospective study being conducted after a considerable length of time, differences in the proficiency of surgeons and the selection of surgical methods may have resulted in biased results. The benefit of surgery in elderly patients with renal cancer remains controversial. This retrospective study aimed to explain the perioperative complications and survival of these patients, to further understand if the surgery benefits. Different from previous studies, the enrolled patients in this study were all elderly patients aged ≥ 75 years, and the conclusions were drawn after grouping analysis to further clarify the advantages and disadvantages of surgery, especially complications using the CDC system.

In conclusion, our study suggests that the overall safety of surgical treatment for elderly patients with renal cell carcinoma is satisfactory. PN should be carefully considered for patients aged ≥ 80 years, as the incidence of intraoperative and postoperative complications is relatively high. It was not to reduce the use of partial nephrectomy in elderly patients, it could be helpful in evidence-based clinical decision-making but should be critically interpreted based on an assessment of the complexity of the tumor and patient's physical condition such as age, underlying diseases and other conditions, technical feasibility, and balance between benefits and risks. Nevertheless, further research and data are needed to strengthen many aspects of the evidence base.

## Data Availability

All of the data are from the National Cancer Center/National Clinical Research Center for Cancer/Cancer Hospital, Chinese Academy of Medical Sciences and Peking Union Medical College. The datasets generated and analysed during the current study are not publicly available due to patient privacy issues (The raw data includes the patient’s name and phone number) but are available from the corresponding author on reasonable request (Dong Wang mobile phone: + 86 1301017215; E-mail: wangdong1199@126.com).

## References

[1] Siegel RL, Miller KD, Fuchs HE, Jemal A (2021). Cancer statistics, 2021. CA Cancer J Clin.

[2] Zheng RS, Sun KX, Zhang SW (2019). Report of cancer epidemiology in China, 2015. Zhonghua Zhong Liu Za Zhi.

[3] Finelli A, Ismaila N, Russo P (2017). Management of small renal masses: American Society of Clinical Oncology clinical practice guideline summary. J Oncol Pract.

[4] Clavien PA, Sanabria JR, Strasberg SM (1992). Proposed classification of complications of surgery with examples of utility in cholecystectomy. Surgery.

[5] Dindo D, Demartines N, Clavien PA (2004). Classification of surgical complications: a new proposal with evaluation in a cohort of 6336 patients and results of a survey. Ann Surg.

[6] Mothes AR, Mothes HK, Radosa MP, Runnebaum IB (2015). Systematic assessment of surgical complications in 438 cases of vaginal native tissue repair for pelvic organ prolapse adopting Clavien–Dindo classification. Arch Gynecol Obstet.

[7] Poletajew S, Zapala L, Piotrowicz S, Wolyniec P, Sochaj M, Buraczynski P (2014). Interobserver variability of Clavien–Dindo scoring in urology. Int J Urol.

[8] Kidney Cancer, Version 2.2022, NCCN clinical practice guidelines in oncology. J Natl Compr Cancer Netw 2022;15(6).

[9] Ljungberg B, Albiges L, Abu-Ghanem Y (2019). European Association of Urology guidelines on renal cell carcinoma: the 2019 update. Eur Urol.

[10] Alam R, Patel HD, Osumah T, Srivastava A, Gorin MA, Johnson MH (2019). Comparative effectiveness of management options for patients with small renal masses: a prospective cohort study. BJU Int.

[11] Becker F, Siemer S, Humke U, Hack M, Ziegler M, Stöckle M (2006). Elective nephron sparing surgery should become standard treatment for small unilateral renal cell carcinoma: long-term survival data of 216 patients. Eur Urol.

[12] Quivy A, Daste A, Harbaoui A (2013). Optimal management of renal cell carcinoma in the elderly: a review. Clin Interv Aging.

[13] Chung JS, Son NH, Lee SE (2015). Overall survival and renal function after partial and radical nephrectomy among older patients with localised renal cell carcinoma: a propensity-matched multicentre study. Eur J Cancer.

[14] Lane B, Campbell SC (2010). Is radical nephrectomy overused in elderly kidney cancer patients?. Aging Health.

[15] Schmitges J, Trinh QD, Sun M (2012). Higher perioperative morbidity and in-hospital mortality in patients with end-stage renal disease undergoing nephrectomy for non-metastatic kidney cancer: a population-based analysis. BJU Int.

[16] Larcher A, Fossati N, Tian Z (2016). Prediction of complications following partial nephrectomy: implications for ablative techniques candidates. Eur Urol.

[17] Tan HJ, Chamie K, Daskivich TJ, Litwin MS, Hu JC (2016). Patient function, long-term survival, and use of surgery in patients with kidney cancer. Cancer.

[18] Harano M, Eto M, Yokomizo A, Tatsugami K, Hamaguchi M, Naito S (2008). The efficacy of laparoscopic radical nephrectomy for renal cell cancer in the elderly: an oncological outcome analysis. Int J Urol.

[19] Berdjis N, Hakenberg OW, Novotny V, Froehner M, Wirth MP (2006). Treating renal cell cancer in the elderly. BJU Int.

[20] Staehler M, Haseke N, Stadler T (2008). Renal surgery in the elderly: morbidity in patients aged > 75 years in a contemporary series. BJU Int.

[21] McKibben MJ, Smith AB (2015). Evaluation and management of the geriatric urologic oncology patient. Curr Geriatr Rep.

[22] Pierorazio PM, Johnson MH, Patel HD (2016). Management of renal masses and localized renal cancer: systematic review and meta-analysis. J Urol.

[23] Rivero JR, De La Cerda J, Wang H (2018). Partial nephrectomy versus thermal ablation for clinical stage T1 renal masses: systematic review and metaanalysis of more than 3900 patients. J Vasc Interv Radiol.

[24] Zabell J, Demirjian S, Lane BR (2018). Predictors of long-term survival after renal cancer surgery. J Urol.

[25] Shuch B, Hanley JM, Lai JC (2014). Adverse health outcomes associated with surgical management of the small renal mass. J Urol.

[26] Tomaszewski JJ, Uzzo RG, Kutikov A (2014). Assessing the burden of complications after surgery for clinically localized kidney cancer by age and comorbidity status. Urology.

[27] Shuch B, Hanley J, Lai J, Vourganti S, Kim SP, Setodji CM (2013). Urologic Diseases in America Project. Overall survival advantage with partial nephrectomy: a bias of observational data?. Cancer.

[28] Lane BR, Fergany AF, Weight CJ (2010). Renal functional outcomes after partial nephrectomy with extended ischemic intervals are better than after radical nephrectomy. J Urol.

[29] Mariusdottir E, Jonsson E, Marteinsson VT (2013). Kidney function following partial or radical nephrectomy for renal cell carcinoma: a population-based study. Scand J Urol.

[30] Medina-Polo J, Romero-Otero J, Rodríguez-Antolín A (2011). Can partial nephrectomy preserve renal function and modify survival in comparison with radical nephrectomy?. Scand J Urol Nephrol.

[31] Reix B, Bernhard JC, Patard JJ (2018). Overall survival and oncological outcomes after partial nephrectomy and radical nephrectomy for cT2a renal tumors: a collaborative international study from the French kidney cancer research network UroCCR. Prog Urol.

[32] Pierorazio PM, Johnson MH, Patel HD (2016). Management of renal masses and localized renal cancer: systematic review and meta-analysis. J Urol.

[33] Antonelli A, Ficarra V, Bertini R (2012). Elective partial nephrectomy is equivalent to radical nephrectomy in patients with clinical T1 renal cell carcinoma: results of a retrospective, comparative, multi-institutional study. BJU Int.

[34] Milonas D, Skulčius G, Baltrimavičius R (2013). Comparison of long-term results after nephronsparing surgery and radical nephrectomy in treating 4- to 7-cm renal cell carcinoma. Medicina (Kaunas).

[35] Minervini A, Serni S, Tuccio A (2012). Simple enucleation versus radical nephrectomy in the treatment of pT1a and pT1b renal cell carcinoma. Ann Surg Oncol.

[36] Tan HJ, Daskivich TJ, Shirk JD, Filson CP, Litwin MS, Hu JC (2017). Health status and use of partial nephrectomy in older adults with early-stage kidney cancer. Urol Oncol.

[37] Bilimoria KY, Liu Y, Paruch JL (2013). Development and evaluation of the universal ACS NSQIP surgical risk calculator: a decision aid and informed consent tool for patients and surgeons. J Am Coll Surg.

